# Use of Robotic Devices in Upper Limb Rehabilitation for Adult Stroke Patients: A Critical Appraisal

**DOI:** 10.1002/hsr2.73153

**Published:** 2026-08-30

**Authors:** Lehan Chu, Jiahui Zou, Shan Liu, Jie Hao

**Affiliations:** ^1^ School of Rehabilitation Sciences and Engineering University of Health and Rehabilitation Sciences Qingdao Shandong China

Dear Editor,

We read with interest the umbrella review protocol by Carrión‑Téllez et al. [1], which aims to synthesize evidence on robotic devices for upper limb rehabilitation after stroke. While the topic is clinically important, several methodological and conceptual issues warrant clarification, as they may affect the validity of the planned review. We would like to raise the following six concerns.

First, the eligibility criteria include robotic devices, exoskeletons, and end‐effector robots targeting upper limb rehabilitation outcomes. However, robotic rehabilitation is a heterogeneous intervention category involving substantially different technologies, including EMG‐driven robotic systems, VR‐based robotic systems, wearable exoskeletons, and assist‐as‐needed systems. Pooling these technologies under a single category of “robotic devices” risks generating conclusions with limited clinical applicability. A more appropriate approach would be to prespecify device classification frameworks and analyze outcomes according to robotic technology type.

Second, the search strategy presented in the protocol appears insufficiently comprehensive for an umbrella review. The authors limited the intervention concept to “robot* OR exoskeleton” and the outcome concept to “upper extremity.” However, terminology in robotic rehabilitation is highly heterogeneous, and relevant systematic reviews may use alternative terms including “robot‐assisted therapy,” “robotic rehabilitation,” “end‐effector devices,” “wearable robots,” “arm function,” “motor recovery,” or “hand function.” Similarly, the population search strategy using only “adult AND stroke” may fail to capture studies indexed using related terms such as cerebrovascular accident, poststroke, hemiparesis, or stroke survivors. Given that umbrella reviews aim to comprehensively synthesize existing evidence, a more sensitive search strategy incorporating broader controlled vocabulary (e.g., MeSH/Emtree terms) and free‐text synonyms would improve retrieval completeness and reduce the risk of selection bias.

Third, the authors' claimed sub‑objectives are not supported by their data extraction plan. The authors claim that their review will provide “a detailed description of the most commonly used robotic devices” and “examine the benefits of their use in combination with other therapeutic modalities.” However, the data extraction items described in the protocol include only standard elements such as authors, year, number of studies, patient numbers, intervention/comparison, design, quality assessment tool, and whether a meta‑analysis was conducted. There are no fields for device type, device parameters, or coding rules for concomitant therapies. If these variables are not systematically extracted, the promised analyses would lack a data foundation. I recommend that the authors publish the full extraction template in the appendix, add the corresponding fields, and clearly define what constitutes “combination therapy” and how it differs from adjunctive interventions in usual care.

Fourth, the protocol does not address the “double amplification” of publication bias that is unique to umbrella reviews. Umbrella reviews use systematic reviews as the unit of analysis. If certain negative primary studies have never been included in any systematic review, their absence becomes completely invisible at the umbrella review level. In this context, pooled conclusions based on multiple systematic reviews may overestimate intervention effects [2]. The protocol does not describe how to identify or handle this issue. I recommend that during data extraction, the authors examine the reference lists of all included systematic reviews to identify whether certain negative primary studies are systematically omitted, and discuss the potential impact of this bias on the review's conclusions.

Fifth, although the authors state that findings will be presented narratively and in tables, the protocol does not provide a detailed plan regarding how evidence from multiple systematic reviews will be synthesized. Given the presence of inconsistent conclusions among previous reviews, a prespecified approach is needed to reconcile discordant findings, integrate effect estimates from existing meta‐analyses, and determine the certainty and clinical relevance of evidence. Furthermore, while AMSTAR‐2 will be used to assess methodological quality of included reviews, it does not evaluate certainty of evidence or strength of clinical recommendations. Incorporating an evidence grading framework would substantially enhance the interpretability of the umbrella review findings.

Sixth, the protocol lacks a hierarchical classification framework for outcome measures. According to the data extraction plan described in the protocol, the authors intend to extract outcomes including motor function, muscle tone, muscle strength, joint range of motion, activities of daily living, among others, and plan to categorize results accordingly. However, this approach does not differentiate the functional levels represented by these measures. Employing the International Classification of Functioning, Disability and Health (ICF) framework to classify outcomes into the three dimensions of body functions, activities, and participation would more clearly reveal the differential efficacy across levels [3]. Mixing outcome measures at the body functions level with those at the activity level without a prespecified hierarchical framework may obscure the critical issue of cross‑level translational efficacy, ultimately failing to determine at which functional level robotic rehabilitation provides genuine benefit. We suggest that the authors add ICF‑based coding fields for outcome measures during data extraction, so that efficacy at different levels can be distinguished in the synthesis, thereby identifying evidence translation gaps.

In conclusion, we commend the authors for addressing a relevant topic, but recommend addressing the above methodological concerns—particularly the lack of an ICF‐based outcome stratification—to improve the validity and clinical utility of the planned review.

## Author Contributions

**Lehan Chu:** conceptualization, methodology, writing – original draft, writing – review and editing. **Jiahui Zou:** writing – original draft, writing – review and editing. **Shan Liu:** methodology, supervision, writing – review and editing. **Jie Hao:** Conceptualization; methodology; writing – original draft; writing – review and editing.

## Funding

The authors have nothing to report.

## Conflicts of Interest

The authors declare no conflicts of interest.

## Transparency Statement

The authors confirm that the results and interpretations presented in this letter are transparent, and the methodology used in the study is clearly documented. All analyses were conducted without manipulation or selective reporting.

## Data Availability

Data sharing is not applicable to this article as no datasets were generated or analyzed during the current study.
